# An Epidemiology Study of Adult Immune Thrombocytopenia Patients in a Teaching Hospital in Northeastern Malaysia

**DOI:** 10.7759/cureus.48533

**Published:** 2023-11-08

**Authors:** Muhamad Aidil Zahidin, Nurul Izzah Abdul Razak, Noor Haslina Mohd Noor, Muhammad Farid Johan, Zefarina Zulkafli, Abu Dzarr Abdullah, Hisham Atan Edinur

**Affiliations:** 1 Department of Hematology, Universiti Sains Malaysia School of Medical Sciences, Kota Bharu, MYS; 2 Department of Internal Medicine, Universiti Sains Malaysia School of Medical Sciences, Kota Bharu, MYS; 3 Department of Forensic Science, Universiti Sains Malaysia School of Health Sciences, Kota Bharu, MYS

**Keywords:** itp, systemic lupus erythematosus, incidence, platelet count, platelet disorder, autoimmune disease

## Abstract

Immune thrombocytopenia (ITP) has been comprehensively studied and understood in Western Europe and various Asia-Pacific regions. However, the epidemiological and clinical-laboratory aspects of ITP in Malaysia remain limited and not well documented. Therefore, this study aims to evaluate the incidence and clinical parameters of ITP using 20-year retrospective data. Medical records for 205 consecutive adult patients with ITP between January 2000 and December 2022 were analyzed. A p-value of <0.05 is considered statistically significant. The majority were Malays (n=192, 93.7%) and females (n=150, 73.2%), with a male-to-female ratio of 1:2.73. One hundred thirty-two (64.4%) and 73 (35.6%) patients were diagnosed with primary ITP (pITP) and secondary ITP (sITP), respectively. Systemic lupus erythematosus (SLE) (n=23, 35.9%), antiphospholipid syndrome (APS) (n=5, 7.8%), and familial thrombocytopenia (n=5, 7.8%) were the top 3 comorbid conditions for ITP. The overall incidence was 1.80/100,000 person-years (95% confidence interval (CI): 1.56-2.07), and the incidences were higher in females than in males with pITP (1.78/100,000 person-years versus 0.70/100,000 person-years) and sITP (0.86/100,000 person-years versus 0.26/100,000 person-years). The median age for patients with pITP was significantly higher than for those with sITP (median: 44 versus 37 years, respectively) (p=0.026). However, there was no statistically significant difference in white blood cell (WBC) counts, hemoglobin (HB) counts, platelet (PLT) counts, absolute neutrophil counts (ANC), or hematocrit (HCT) counts between those with pITP and sITP at the time of diagnosis. The current study provides an overview of ITP epidemiology in northeastern Malaysia. We emphasize the critical need for further additional research, particularly at the state and national levels in the future.

## Introduction

Immune thrombocytopenia (ITP) is an acquired autoimmune disorder characterized by a low platelet count of less than 100×10⁹/L [1]. The etiology of ITP mainly involves antiplatelet autoantibodies; however, other factors and mechanisms have also been observed to contribute to the pathophysiology of ITP, including viral infections, genetics, medications, and other related conditions [2-4].

ITP can be categorized into several classifications based on specific characteristics. Primary ITP (pITP), the most prevalent form of this condition, is characterized by low platelet production and premature platelet breakdown, which primarily results from the presence of antiplatelet autoantibodies [5]. Furthermore, secondary ITP (sITP) can arise from underlying conditions such as viral infections, autoimmune disease, medication, or cancer [1,2]. According to the 2019 American Society of Hematology (ASH) guidelines on ITP, there are several phases of ITP based on the duration of the illness. Newly diagnosed or acute ITP occurs within the first three months and generally affects children. Persistent ITP can last between three to 12 months, while chronic ITP can continue for more than 12 months and is often diagnosed in adult patients [5]. Diagnosing ITP can be a complex procedure mainly reliant on the exclusion of other potential causes, as there is no gold standard test that can precisely verify the diagnosis. Basic evaluations such as patient and family history, physical examination, full blood count (FBC), bone marrow examination, *Helicobacter pylori* testing, and immunological testing have been used to support the diagnosis [6,7].

In general, research on ITP has undergone a drastic change over the years in Western Europe and East Asian regions, particularly regarding the basic clinical course of the disease [2,8-15]. However, there remains a lack of adequate clinical epidemiology and laboratory data, considering that the only epidemiology studies in Malaysia were conducted on the west coast of Peninsular Malaysia, in Perak and Selangor [9,14]. The limited research conducted in various parts of Malaysia can significantly limit the understanding of the distribution and determinants of diseases within a population. Therefore, this retrospective study was conducted to determine the clinical epidemiology of adult ITP patients in northeastern Malaysia.

## Materials and methods

Study design

The study comprised 205 adult patients diagnosed with pITP and sITP from January 2000 to December 2022 at Hospital Universiti Sains Malaysia. All patient data were accessed from the Haematology Patient Information System (HPIS) and Lab Information System (LIS) databases and tabulated in Microsoft Excel (Microsoft Corporation, Redmond, WA). The study population included all ITP patients aged 18 years and older with a platelet count of <100×10^9^/L. ITP was defined based on the International Classification of Diseases version 11 (ICD-11:3B64) and the Malaysian Clinical Practice Guidelines [7,16].

Patients with pITP were diagnosed by excluding other causes of thrombocytopenia through their medical history, physical examination, and routine hematological tests such as FBC and peripheral blood film. Comorbid diseases were identified and tabulated using ICD-11 criteria, such as systemic lupus erythematosus (SLE) (ICD-11:4A40.0), antiphospholipid syndrome (APS) (ICD-11:4A45), autoimmune hemolytic anemia (AIHA) (ICD-11:3A20), *H. pylori* infection (ICD-11:XN3DY), hepatitis B virus (HBV) (ICD-11:XN0GA), and hepatitis C virus (HCV) (ICD-11:XN1EZ). The differential diagnosis involved autoimmune screening (e.g., antinuclear antibody, anticardiolipin antibody, direct platelet immunofluorescence test, and platelet membrane glycoprotein antibodies) for common autoimmune diseases, as well as bacterial and viral testing (e.g., *H. pylori*, HIV, HBV, and HCV) for patients at risk of infection.

Data collection

Demographic data (e.g., gender, age, and ethnicity) and clinical characteristics (e.g., white blood cell (WBC) counts, hemoglobin (HB) counts, platelet (PLT) counts, absolute neutrophil counts (ANC), and hematocrit (HCT) counts) at the initial diagnosis of ITP were abstracted from the patient's medical records.

Statistical analysis

Demographic data with categorical variables were presented as frequencies and percentages, while numerical variables were presented as median with interquartile range (IQR). The incidence rate was calculated with 95% confidence intervals (CIs). A stratified association analysis was conducted, considering gender (e.g., male and female), ethnicity (e.g., Malay, Chinese, and Indian), age (e.g., <30 and ≥30 years), and hematological parameters (e.g., WBC, HB, PLT, ANC, and HCT) between those with pITP and those with sITP. The Kolmogorov-Smirnov test was employed for the normality test. The Pearson chi-square test and Mann-Whitney U test were calculated using the Statistical Package for the Social Sciences (SPSS) version 27 (IBM SPSS Statistics, Armonk, NY). Data were considered statistically significant at p<0.05.

Ethical consideration

This retrospective study was reviewed and approved by the Human Research Ethics Committee of Universiti Sains Malaysia (USM/JEPeM/19090533).

## Results

A total of 205 patients were diagnosed with ITP at Hospital Universiti Sains Malaysia between January 2000 and December 2022. The demographic data of the ITP patients are summarized in Table 1. The male-to-female ratio was 1:2.73 (males: n=55, 26.8%; females: n=150, 73.2%), and the age range was between 19 and 90 years. The majority of the patients were Malays (n=192, 93.7%), followed by Chinese (n=12, 5.9%) and Indians (n=1, 0.5%). Overall, the incidence of ITP was 1.80/100,000 person-years (95% CI: 1.56-2.07). It was higher in females (2.64/100,000 person-years, 95% CI: 2.23-3.10) than in males (0.97/100,000 person-years, 95% CI: 0.73-1.26).

**Table 1:** Demographics of the 205 ITP patients ITP: immune thrombocytopenia, IQR: interquartile range, SLE: systemic lupus erythematosus, AIHA: autoimmune hemolytic anemia, APS: antiphospholipid syndrome, ALL: acute lymphoblastic leukemia, HCV: hepatitis C virus, Evans: Evans syndrome, Graves': Graves' disease, COVID-19: coronavirus disease 2019

	Number	%
Gender		
Male	55	26.8
Female	150	73.2
Age (years)		
Age range	19-90	
Mean age (IQR)	42 (33-58)	
Ethnicity		
Malay	192	93.7
Chinese	12	5.9
Indian	1	0.5
Type of ITP		
Primary	141	68.8
Secondary	64	31.2
Phase of ITP		
Acute	51	24.9
Persistent	24	11.7
Chronic	130	63.4
Comorbid		
SLE	23	35.9
APS	5	7.8
Familial thrombocytopenia	5	7.8
HCV	4	6.3
AIHA	3	4.7
Drug induced	3	4.7
Breast cancer	2	3.1
Myelodysplastic syndrome	2	3.1
Autoimmune hepatitis	1	1.6
Autoimmune thyroid	1	1.6
Colon cancer	1	1.6
Dengue fever	1	1.6
Evans syndrome	1	1.6
Graves' disease	1	1.6
Helicobacter pylori	1	1.6
Hepatitis B virus	1	1.6
Concurrent diseases		
SLE+AIHA	2	3.1
SLE+APS	2	3.1
Evans+HCV	1	1.6
Graves'+COVID-19	1	1.6
SLE+APS+AIHA	1	1.6
SLE+drug induced	1	1.6
SLE+Evans	1	1.6

One hundred forty-one (68.8%) and sixty-four (31.2%) patients were diagnosed with pITP and sITP, respectively. A total of 16 types of diseases were comorbid with ITP (Table 1). Of those diagnosed with sITP, 23 had SLE (35.9%), five had APS and familial thrombocytopenia (7.8%), four had HCV (6.3%), three had AIHA and drug-induced (4.7%), and two had breast cancer and myelodysplastic syndrome (3.1%). Meanwhile, the other comorbidities were reported as single cases. One (1.6%) individual was reported to have three concurrent autoimmune diseases: SLE, APS, and AIHA. Two concurrent diseases (3.1%), SLE+AIHA and SLE+APS, were also recorded among ITP patients, each with two cases.

The overall incidence for pITP and sITP was 1.24/100,000 person-years (95% CI: 1.04-1.46) and 0.56/100,000 person-years (95% CI: 0.43-0.71), respectively. In terms of gender, the incidence was higher for females than for males with both pITP (1.78/100,000 person-years, 95% CI: 1.45-2.16 versus 0.70/100,000 person-years, 95% CI: 0.50-0.96) and sITP (0.86/100,000 person-years, 95% CI: 0.64-1.14 versus 0.26/100,000 person-years, 95% CI: 0.15-0.43).

Table 2 shows the demographic and clinical characteristics of those with pITP and sITP. The age of pITP patients (median: 44 years, IQR: 34-61.5 years) was significantly older than that of sITP patients (median: 37 years, IQR: 31-53 years) (p=0.026). There was no statistically significant difference in terms of gender and hematological parameters (WBC, HB, PLT, ANC, and HCT) at the time of diagnosis.

**Table 2:** Demographic and clinical characteristics of pITP and sITP patients at the early diagnosis ITP: immune thrombocytopenia, WBC: white blood cell counts, HB: hemoglobin counts, PLT: platelet counts, ANC: absolute neutrophil counts, HCT: hematocrit counts, IQR: interquartile range

	Primary ITP (n=141)	Secondary ITP (n=64)	Total (n=205)	p-value
Gender				
Male	40 (28.4%)	15 (23.4%)	55 (26.8%)	0.460
Female	101 (71.6%)	49 (76.6%)	150 (73.2%)	
Ethnicity				
Malay	131 (92.9%)	61 (95.3%)	192 (93.7%)	0.624
Chinese	9 (6.4%)	3 (4.7%)	12 (5.9%)	
Indian	1 (0.7%)	0 (0%)	1 (0.5%)	
Age (years)				
<30	18 (12.8%)	10 (15.6%)	28 (13.7%)	0.581
≥30	123 (87.2%)	54 (84.4%)	177 (86.3%)	
Median (IQR)	44 (34-61.5)	37 (31-53)	42 (33-58)	0.026
PLT (×10^9^/L)				
0-10	37 (26.2%)	17 (26.6%)	54 (26.3%)	0.654
11-30	34 (24.1%)	19 (29.7%)	53 (25.9%)	
≥30	70 (49.6%)	28 (43.8%)	98 (47.8%)	
Median (IQR)	30 (10-59)	23 (10-59.5)	27 (10-59)	0.788
Other parameters (median (IQR))				
WBC (×10^9^/L)	8.6 (6.5-10.7)	8.6 (6.5-11.1)	8.6 (6.5-10.8)	0.832
HB (g/dL)	12.2 (11-13.8)	12.4 (10.7-13.3)	12.3 (10.9-13.8)	0.614
ANC (×10^9^/L)	4.8 (3.4-6.9)	5.6 (4-7.3)	4.9 (3.6-6.8)	0.545
HCT (%)	35 (29.3-40.8)	36.1 (32.6-40.5)	36 (30-40.6)	0.427

## Discussion

Clinical epidemiology is crucial in determining the primary etiologies and appropriate treatment for each patient. However, ITP in Malaysia is still underexplored and has not been systematically studied, resulting in insufficient information on the disease, especially in clinical epidemiology and laboratory data. The first study on ITP in Malaysia was conducted in the 1980s [17], yet epidemiology studies in the country only began to be published in 2021 [9], with a focus on the west coast of Peninsular Malaysia. Analysis of the clinical epidemiology and laboratory aspects of ITP can contribute to the diagnosis and optimization of patient care, besides providing direction for further studies on the pathophysiology and the development of a gold standard test. Given Malaysia's diverse population, which includes people of various ethnicities and genetic backgrounds [18], more research is needed to understand potential differences in the prevalence and clinical presentation of ITP among different demographic groups. Therefore, conducting a study in the northeastern Malaysian population can provide a more comprehensive picture of the variation of the clinical pathogenicity of ITP in Malaysia.

We identified that the male-to-female ratio of ITP patients in Kelantan is within the range of previous studies done in Selangor (1:2.5) and Perak (1:3) [9,14]. In contrast, a lower male-to-female ratio was reported in studies from India (1:1.9) and Western countries such as France (1:1.5) and the United Kingdom (1:1.45) [2,19,20]. Furthermore, our study noted a significantly higher ITP incidence rate among females compared to males, which is comparable with previous studies from the United Kingdom (58.22/100,000 versus 40.66/100,000 person-years) and France (3.03/100,000 versus 2.77/100,000 person-years) [2,20]. Additionally, local research also observed higher ITP rates among female young adults (<65 years old) [14].

A study by Invernizzi et al. reported that females accounted for approximately 80% of all autoimmune disease patients [21]. The differences in gender-related immune responses contribute to the high incidence of autoimmune diseases among females, which includes the role of the X chromosome and the regulation of sex hormones in the immune system [22,23]. Incomplete activation of two copies of the X-linked gene can lead to the production of functional proteins with downstream effects [24]. Sex hormones such as estrogen have also been found to be associated with the regulation of autoimmunity [25]. The immunostimulatory function of estrogen increases susceptibility to autoimmune diseases such as SLE [26]. A previous study reported that a high level of estrogen can inhibit the expression of pro-inflammatory cytokines, thereby reducing the risk of rheumatoid arthritis and multiple sclerosis [25].

Our study confirms that the prevalence of Malay ITP patients is the highest compared to other ethnic groups (Chinese and Indian), as reported by previous local studies in Perak and Selangor [9,14]. About 63% of the total population in Peninsular Malaysia is constituted by Malays [18], thus establishing their dominance in the research.

In this retrospective study, we also conducted an extensive comparison of ITP incidence rates within the country with those of global general populations (Table 3). Thus, we calculated the incidence rates in Perak [9] and Selangor [14] using the demographic data provided by the Department of Statistics of Malaysia [27]. In summary, the incidence of ITP in Peninsular Malaysia registers at less than 10 cases per 100,000 person-years. Notably, Perak exhibits a higher incidence rate at 9.61/100,000 person-years, surpassing Kelantan (this study) and Selangor (0.76/100,000 person-years). The higher incidence observed in Perak is likely attributed to variations in the study's time frame, encompassing one year, compared to the broader time span of 10-20 years for both Selangor and the present study.

**Table 3:** Summary of the incidence rate of ITP in the general population ITP: immune thrombocytopenia

Year	Author	Country	Incidence rate (per 100,000 person-years)
2023	This study	Kelantan, Malaysia	1.80
2022	Hamzah et al. [14]	Selangor, Malaysia	0.76
2021	Sulaiman et al. [9]	Perak, Malaysia	9.61
2018	Hung et al. [13]	Taiwan	2.00
2017	Lee et al. [11]	Korea	1.50
2014	Moulis et al. [2]	France	2.94
2010	Kurata et al. [12]	Japan	2.16
2010	Terrell et al. [8]	Europe	3.30
2009	Schoonen et al. [10]	England	3.90
1999	Frederiksen et al. [15]	Denmark	2.68

Across Asia, the incidence of ITP falls within the range of 1.50/100,000 to 2.16/100,000 person-years [11-13]. Conversely, in Europe, the incidence is slightly higher, ranging from 2.69/100,000 to 4.00/100,000 person-years. The disparities in ITP incidence rates across Malaysia, Asia, and Europe could be attributed to various factors, including genetic predisposition, healthcare practices, and overall population demographics. Genetic predispositions play a significant role, as different populations may have varying genetic susceptibilities to ITP [28,29]. Additionally, variations in healthcare practices, access to medical care, and diagnostic capabilities may significantly influence the reporting and identification of ITP cases. Demographic differences, such as age, gender, and racial distribution, also play a pivotal role in contributing to the observed variations in ITP incidence both between and within these continents.

The pathophysiology of ITP is multifactorial and can overlap with other underlying conditions, including autoimmune and infectious diseases [3,5]. Our study revealed that at least 30% of overall ITP patients were associated with comorbid diseases, and 50% of the cases accounted for autoimmune diseases such as SLE, APS, and AIHA, while viral diseases and cancer represented less than 10% each. A study in the United Kingdom reported a high percentage (76%) of association between autoimmune diseases and sITP, with SLE being the most common autoimmune disease comorbid with ITP [10]. A local cohort study by Sulaiman et al. also discovered a significant association between SLE and sITP, despite the small number of reported cases [9].

The median PLT count for pITP is slightly higher than that for sITP (median: 30×10⁹/L (IQR: 10-59×10⁹/L) versus 23×10⁹/L (IQR: 10-59×10⁹/L). Even so, no significant difference was found between both groups. In contrast with previous studies in Pakistan, sITP patients have a higher mean ± standard deviation (SD) than pITP patients (54.1±26.9 ×10⁹/L versus 31.5±21.9×10⁹/L) [30]. A significantly high median initial PLT count was observed in patients with sITP (60×10⁹/L) compared to patients with pITP (3.5×10⁹/L) in previous research conducted in Jordan [4]. The other hematological parameters, such as WBC, HB, ANC, and HCT, showed no significant difference between patients with pITP and sITP. This is in contrast with the previous study in Pakistan, where most of the hematological parameters, including WBC, HB, HCT, and PLT, showed significant differences between those with pITP and those with sITP [30].

This retrospective study was conducted at a single-center hospital in Kota Bharu district. Therefore, the epidemiological findings from this study may not apply to the broader ITP population, encompassing both untreated individuals and those who have never been hospitalized. These individuals are typically asymptomatic and/or may have exhibited mild thrombocytopenia. Our hospital is located within a high-density population comprising three main ethnicities: Malays, Chinese, and Indians. Hence, the occurrence and frequency of ITP among Orang Asli (indigenous people) and other ethnic populations might be underestimated.

## Conclusions

In conclusion, this retrospective study demonstrates clinical epidemiology outcomes that are consistent with both local and global studies. Our findings highlight a notable female-to-male ratio, with a higher frequency of ITP observed among patients aged above 30 years old, females, Malays, and those with pITP. SLE was the most diagnosed comorbid disease with ITP. The overall median age for pITP patients was significantly older compared to sITP patients. However, no significant differences were found in terms of gender, ethnicity, PLT counts, and other hematological parameters. This retrospective study underscores the importance of ongoing research into ITP, both at the state and national levels. Considering a multicentered study can enhance our understanding of its epidemiology and etiology while allowing for the generalization of the findings. Moreover, this knowledge may lead to a more comprehensive insight, enabling better diagnostic and treatment strategies.
